# Supplementary material for: Epidemiology, Microbiology and Severity of Bronchiolitis in the First Post-Lockdown Cold Season in Three Different Geographical Areas in Italy: A Prospective, Observational Study
Source: Children (Basel). 2022 Apr 1;9(4):491. doi: 10.3390/children9040491 (PMC9024462; doi:10.3390/children9040491)
Supplement: Supplementary file 1 [file children-09-00491-s001.zip › children-1647085-supplementary.pdf]

**Supplementary Material**

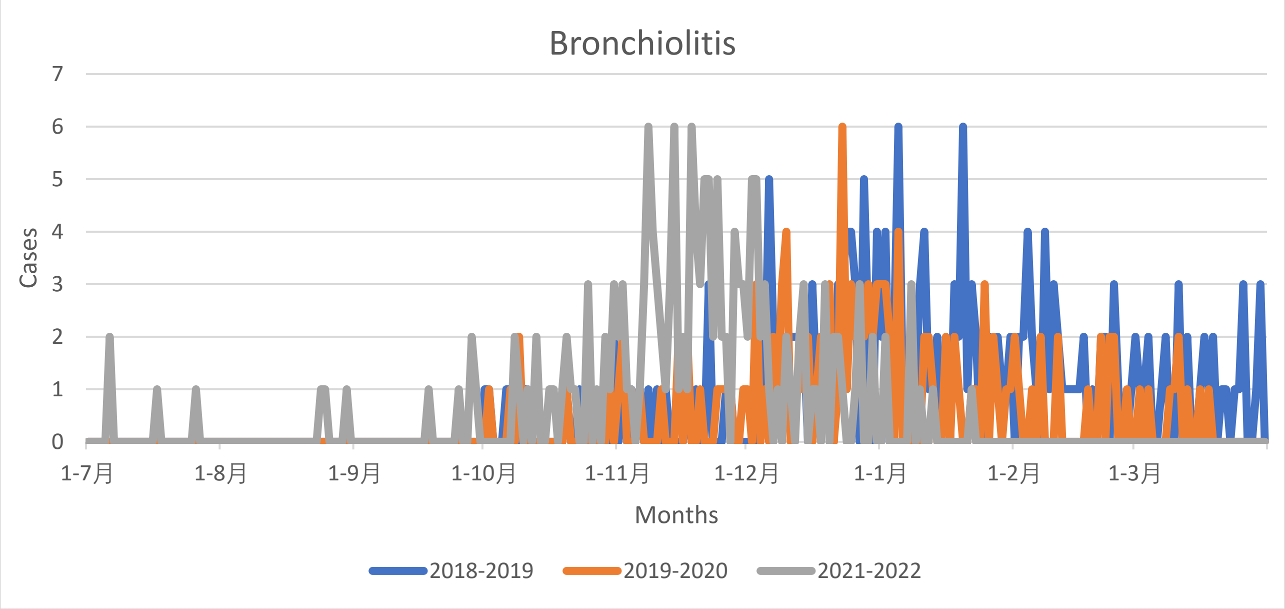


**Figure S1.** Temporal distribution of bronchiolitis cases in Rome (Central Italy), during the 2018-19, 2019-20 and 2021-22 seasons.

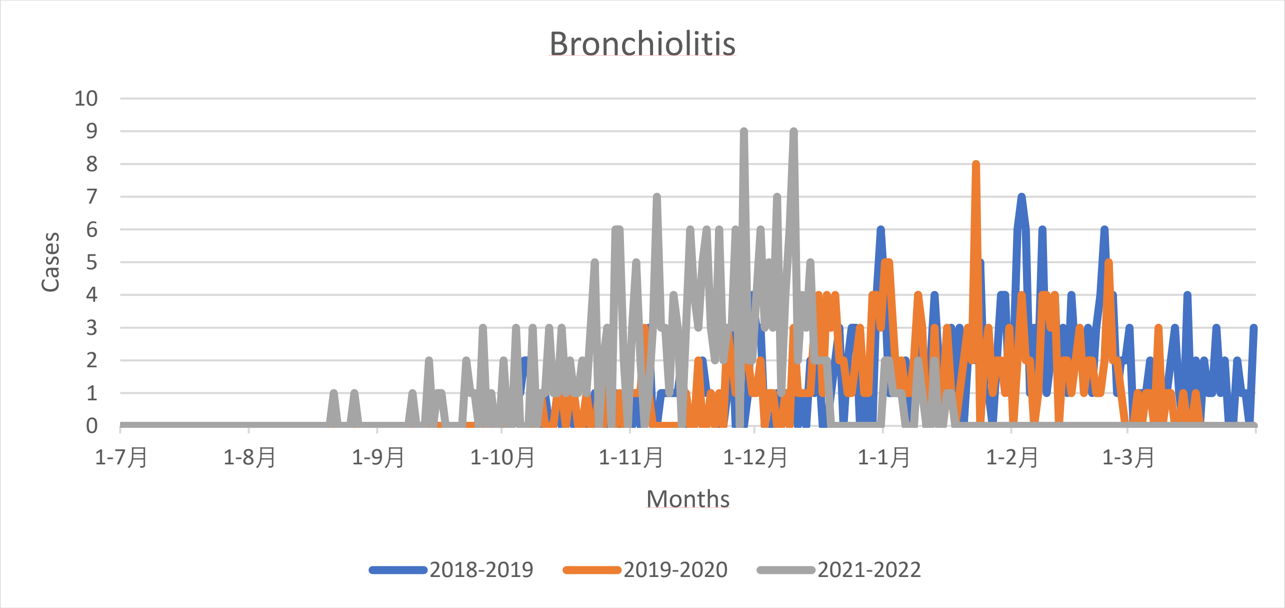


**Figure S2.** Temporal distribution of bronchiolitis cases in Bologna (Northern Italy), during the 2018-19, 2019-20 and 2021-22 seasons.

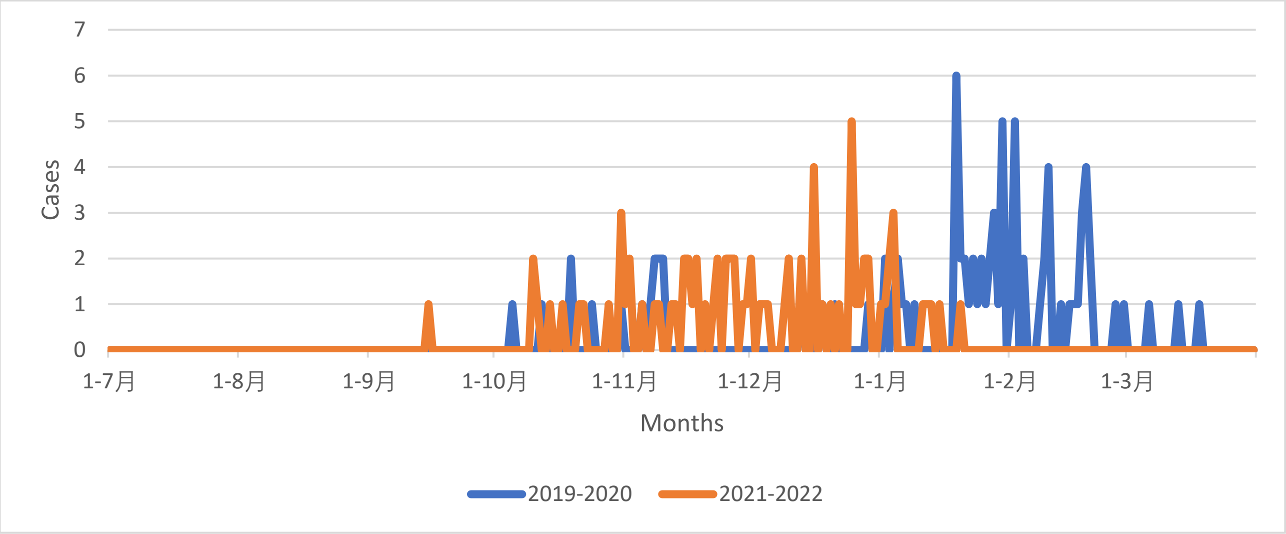


**Figure S3.** Temporal distribution of bronchiolitis cases in Catania (Southern Italy), during the 2019-20 and 2021-22 seasons.
